# The Dominating Role of Genetic Background in Shaping Gut Microbiota of Honeybee Queen Over Environmental Factors

**DOI:** 10.3389/fmicb.2021.722901

**Published:** 2021-11-05

**Authors:** Jiandong Yang, Yun Zhong, Liqun Xu, Bo Zeng, Kang Lai, Mingxian Yang, Diyan Li, Ye Zhao, Mingwang Zhang, Debing Li

**Affiliations:** ^1^College of Animal Sciences and Technology, Sichuan Agricultural University, Chengdu, China; ^2^Sichuan Province Apiculture Management Station, Chengdu, China

**Keywords:** honeybee, queen, gut microbiota, amplicon sequencing, asexual hybridization, crossbreed

## Abstract

A balanced, diverse gut microbiota is vital for animal health. The microbial population is shaped by multiple factors including genetic background and environment, but other determinants remain controversial. Numerous studies suggest that the dominant factor is genetic background while others emphasize the environmental factors. Here, we bred asexual hybridization queens (AHQs) of honeybees through nutritional crossbreeding (laid in *Apis mellifera* colony but bred in *Apis cerana* colony), sequenced their gut microbiome, and compared it with normally bred sister queens to determine the primary factor shaping the gut microbiota. Our results showed that the dominant genera in the gut microbiota of AHQs were *Brevundimonas*, *Bombella*, and *Lactobacillus*, and its microbial community was more related to *A. mellifera* queens. The AHQs had a moderate number of different bacterial species and diversity, but total bacterial numbers were low. There were more significant taxa identified in the comparison between AHQ and *A. cerana* queen according to LEfSe analysis results. The only genetic-specific taxon we figured out was *Brevundimonas*. The growth of core bacterial abundance showed different characteristics among different queen groups in the first week after emerging. Collectively, this study suggested that the genetic background played a more dominant role than environmental factors in shaping the gut microbiota of honeybee queen and the microbiota of midgut was more sensitive than that of rectum to this impact.

## Introduction

Diverse microbial communities colonize different host tissues, with the gut harboring the densest and most diverse range of species (Martinson et al., 2012). Researchers delve into the gut microbiota of animal newborns, which underlines the vital role of the gut microbiota for host’s health by maintaining intestinal homeostasis and barrier function, stimulating the development of the immune system, contributing to nutrient digestion, and protecting against pathogens (Sekirov et al., 2010; Maynard et al., 2012; Wopereis et al., 2014). Current evidence indicates that the gut microbiota of honeybees is pivotal to their health as it participates in metabolism and immunity, promotes development, and resists invasion by parasites and pathogens (Guo et al., 2015; Schwarz et al., 2016; Zheng et al., 2017; Wu et al., 2020). As a result, the functional role of the gut microbiota has drawn much attention worldwide. Besides function, research has been focused on identifying the dominant factors determining the diversity and richness of the gut microbiota. Data from a variety of animal subjects concur that there is a complex interaction between the microbial community and the host, but the primary determinants of the animal gut microbiota include the host’s genotype (Knowles et al., 2019; Korach-Rechtman et al., 2019), diet (David et al., 2014; Carmody et al., 2015; Sonnenburg et al., 2016; Griffin et al., 2017; Jones et al., 2017), season (Ludvigsen et al., 2015), host age (Martinson et al., 2012; Tarpy et al., 2015; Anderson et al., 2018), caste (Kapheim et al., 2015; Anderson et al., 2018), and environment (Amato et al., 2016; Ludvigsen et al., 2017; Ren et al., 2017). Multiple nature and nurture effects stemming from differences in host species can greatly influence the interactions between host and microorganism. Even within the same species, conclusions about the dominant influences can vary because of individual differences and the type of calculation methods used. We are only just beginning to understand the processes shaping the composition of host-associated microbial communities over evolutionary and ecological timescales (Foster et al., 2017).

Honeybees are necessary and valuable pollinators of most crops and wild plants, and their economic value in this sense far outweighs their usefulness as honey producers (Van der Sluijs and Vaage, 2016). Their intestinal organs are segmented, and the composition of the gut microbial community is relatively simple, making it an ideal social insect model for studying the impact of social behavior on the dynamics of the gut microbiota. The detailed taxonomic information about the gut microbiota composition of the honeybee (*Apis mellifera*) remained unavailable until high-throughput sequencing (16S amplicon sequencing) was developed and widely employed (Jeyaprakash et al., 2003). Numerous studies suggested that there was a conserved evolutionary pattern of the gut communities in all related corbiculate (pollen basket) bee species, which could insure they have a similar and relatively stable gut microbial community which mainly contained five core members and four non-core members (Martinson et al., 2011; Sabree et al., 2012; Kwong et al., 2017). These core members comprise a remarkably stable characteristic as they can be detected in the gut of every adult worker, whether in the same region or the same colony. Although there is great variability in the microbial population between each individual worker, these microbes could rarely be found in honeybee living environments, including pupae, frame, and hive (Engel and Moran, 2013; Powell et al., 2014). We believe that this phenomenon indicates that there may have been a strong mutual selection between the gut microbiota and the host during evolution, and the explanation for this phenomenon can be seen from the biological characteristics of the social lifestyle of honeybees (Kwong et al., 2017).

Social behavior is a prominent feature of social animals, one of which is honeybee, and one of their social behaviors is mutual feeding. Newly emerged queens and workers are sterile (Martinson et al., 2012; Powell et al., 2014); they usually stay in hives for more than 1 week after emerging and were fed royal jelly by other nurse bees. Without contacting with the outside environment, their gut microbe can develop rapidly within 5 days. During these 5 days, the core members colonize rapidly and the microbial community gradually forms (Guo et al., 2015). Royal jelly, as the main food for newborns, may play an important intermediary role in the microbial transfer process when mutual feeding happened. Thus, as a social behavior, mutual feeding provides a stable pathway for the transfer of the gut microbiota between individuals and is of great significance for the early growth of core members to occupy key metabolic niches (Powell et al., 2014). This pattern of social transmission can also be found in other social living animals and humans (Marcobal and Sonnenburg, 2012). The reason why these gut core microbes plays irreplaceable roles in the host’s intestinal tract can be explained as that they occupy some vital metabolic niches, such as helping the host to digest pollen and nectar (Zheng et al., 2016), synthesizing hormones (Zheng et al., 2017), and regulating immune responses (Wu et al., 2020). However, recent studies suggest that some of the non-core microbiota may play important roles in caste development because they maintain a high relative abundance in the early developmental stages of the queen (Jeyaprakash et al., 2003; Corby-Harris et al., 2014b; Anderson et al., 2018); the mechanism remains to be characterized.

The Western honeybee, *A. mellifera*, and the eastern honeybee, *Apis cerana*, are the most widely raised honeybee species in China, bringing the most economic income to Chinese beekeepers compared to other bee species. These two bee species have unique biological characteristics and genetic backgrounds and also have excellent individual productive traits. They do have some shortcomings for beekeepers, however. For instance, the eastern bee produces less honey but has strong disease resistance and is easy to manage manually (Li et al., 2012), while the Italian bee is a high-yielder but more likely to be infected by pathogens and parasites (Guo et al., 2015) and requires more keeper management. It would be advantageous to create a bee variety with high yield, high disease resistance, easy feeding, and management to increase the profitability of beekeeping. Chinese researcher Ming Zhuang has created a hybrid bee with the advantages of both parents by transferring (Zhuang, 1985). This method of hybridization, which does not change the genetic background of the offspring, is a form of asexual reproduction called nutritional crossbreeding. Many additional attempts to produce hybrid bees by nutritional crossbreeding have been made by Chinese researchers, and their production and physiological indices have been measured. They saw some changes in morphology (Zeng et al., 2005a), but surprisingly they found that performance parameters such as birth weight (Zeng et al., 2005b) and mite resistance (Xie et al., 2008) of the offspring of the bees produced by nutritional crossbreeding were also improved. The crossbred queen (transferred from colony A to colony B) is an ideal model for identifying the dominant factors shaping the gut microbiota. The genetic background of the crossbred queens is consistent with that of the queens from colony A, while the living conditions and nutritional factors acquired are those of the queens from colony B.

Here, we use the asexual hybridization queen (AHQ) social-animal model to discover the dominant factors influencing the composition of the gut microbiota in the early development stage of honeybee queens, to explore the interaction between the gut microbiota and the host. This study may help to reveal how social living affects the gut microbiota and allow a deeper exploration of its relationship with the host during coevolution.

## Materials and Methods

### Grouping of Queens and Nomenclature

Three groups of queens were involved in this study: (1) ACQs, usual *A. cerana* queens that were laid in an *A. cerana* colony and bred by an *A. cerana* nurse bee; (2) AMQs, usual *A. mellifera* queens that were laid in an *A. mellifera* colony (AMC) and bred by an *A. mellifera* nurse bee; and (3) AHQs, an asexual hybridization (nutritionally crossbred) of queens that were laid in an AMC, but fostered by an *A. cerana* nurse bee.

### Experimental Design and Management

**FIGURE 1:**
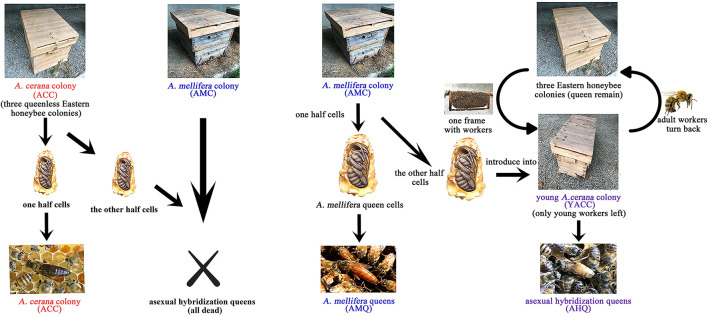
Experimental design. The brief process of ACQs, AHQs, and AMQs included in this study is visualized in this schematic diagram. Experimental details are given in the “Materials and Methods” section. The other opposite crossbreeding (*A. cerana* into *A. mellifera*) was excluded from this study for lack of acceptance.

All the colonies used in this study were located at the affiliated apiculture base of the College of Animal Sciences and Technology, Sichuan Agricultural University, Chengdu, Sichuan, China. The experimental design is shown in Figure 1. Three robust *A. mellifera* colonies were selected to breed AMQs, 3 robust *A. cerana* queen-less colonies were chosen to breed ACQs, and 10 additional robust *A. cerana* colonies were used to organize the young *A. cerana* colonies (YACCs). We prepared and introduced 60 queen cells (30 for breeding AMQs, 30 for breeding AHQs) along with larvae for each AMC and 30 queen cells (all for breeding ACQs) for each *A. cerana* colony using artificial queen-breeding technology. The YACCs were organized on the sixth day after the queen cells were introduced successfully and completely. To organize the YACCs, the bee frames along with workers were taken from the 10 additional robust *A. cerana* colonies and placed one to one into each new sterilized hive, at a distance of 50 m away from the original colonies. Adult workers of recognition capability returned to their original hive leaving newly emerged nurse bees in the YACCs to foster the *A. mellifera* queens after they emerged from the queen cells. Once the YACCs were organized, half of the queen cells were transferred from AMCs to YACCs so that there were 30 queen cells along with queens in each experimental group. Each queen in a hive was checked every morning and marked to record age. In order to set up the opposite crossbreeding, we tried transferring *A. cerana* queen pupae to a young AMC to breed the other kind of crossbred queen but failed with an almost total lack of acceptance. This may be because *A. mellifera* workers have a greater ability to recognize and exclude different species than *A. cerana*. Thus, this reverse asexual hybridization group was not included in the study.

The core members of the gut microbe population of the workers were colonized by the fifth day after their host emerged; however, there have been only limited reports on the exact timing of the establishment of queens’ gut microbes (Guo et al., 2015). Queens usually flew out from the hive and mated with drones at the seventh day after emerging, when their tissues and organs were almost fully developed. Based on this knowledge, gut samples used for high-throughput sequencing were collected when queens were 5 days old while other samples for use real-timequantitative PCR (qPCR) were collected on the first, fourth, and seventh days to monitor the absolute abundance changes of the core members in the queens’ gut microbial community. Due to the high individual diversity of animals gut microbes, we used as many samples as possible for high-throughput sequencing to minimize the impact of such diversity on our conclusions.

### DNA Extraction and qPCR

The queens were first euthanized with carbon dioxide and then pinned in a sterile dissecting plate. The gut tissues were collected by clamping the last part of the sternum with sterilized forceps, separating the midgut and rectum from the gut tissues, and placing them into 2.0-ml microfuge tubes. The gut tissues were immediately frozen in liquid nitrogen and transferred to −80°C until DNA extraction. The entire procedure was conducted under aseptic conditions, and all tools were sterilized. Total genomic DNA was extracted from the midgut and rectum using the TIANamp^®^ Stool DNA Kit (Beijing Tiangen Biotech Ltd., Beijing, China) following the manufacturer’s instruction under sterile conditions as described by Powell et al. (2014). DNA purity was determined on a 1% agarose gel, and DNA was diluted to 1 ng/μl using sterile water. One rectal DNA sample was discarded due to the poor quality.

The 16S rRNA gene V3–V4 regions were amplified using universal primers 341F (5′-CCTAYGGGRBGCASCAG-3′) and 806R (3′-GGACTACNNGGGTATCTAAT-5′) (Wang and Qian, 2009; Thijs et al., 2017) with the barcode. All PCR reactions were carried out with 15 μl of Phusion^®^ High-Fidelity PCR Master Mix (New England Biolabs, Ipswich, MA, United States), 0.2 μM of forward and reverse primers, and about 10 ng of template DNA. Thermocycling was performed with an initial denaturation at 98°C for 1 min, followed by 30 cycles of denaturation at 98°C for 10 s, annealing at 50°C for 30 s, elongation at 72°C for 30 s, and lastly, 72°C for 5 min. PCR amplification products were verified by electrophoresis on a 1% agarose gel. Equal volumes of 1× loading buffer containing SYBR Green (New England Biolabs) were mixed with PCR products in equidensity ratios and separated by electrophoresis on a 2% agarose gel for detection. Then, the PCR products were purified with the Qiagen gel extraction kit (Qiagen, Hilden, Germany).

### Illumina Sequencing and Sequence Analysis

Sequencing libraries were generated using the TruSeq^®^ DNA PCR-Free sample Preparation Kit (Illumina, San Diego, CA, United States) following the manufacturer’s recommendations, and index codes were added. The library quality was assessed on the Qubit 2.0 fluorometer (Thermo Scientific, Waltham, MA, United States) and Agilent Bioanalyzer 2100 system. The libraries were sequenced on an Illumina NovaSeq platform, and 250-bp paired-end reads were generated. Paired-end reads were assigned to samples based on their unique barcode and truncated by cutting off the barcode and primer sequence. All paired-end reads were merged using FLASH software (v1.2.7)^1^ (Magoč and Salzberg, 2011) and entered into QIIME 2 (v2019.7)^2^ (Bolyen et al., 2019) for downstream analysis including demultiplexing, pair joining, de-noising, and clustering. Amplicon sequence variants (ASVs) were defined based on 100% similarity clustering using the deblur (Amir et al., 2017) plugins in QIIME 2. Afterward, the representative sequences of each ASV were aligned and used to generate a phylogenetic tree as a reference for phylogenetic diversity analyses. Lastly, the 16S rRNA gene total length Silva database (v132_99_16S)^3^ was specifically retrained for V3–V4 regions and used to classify the representative sequences.

### Absolute qPCR

Absolute qPCR was used to determine the variation in abundance of the core members of the queens’ gut microbiota. The primers used in this process are listed in Supplementary Table 1, and the initial template DNA concentrations were normalized between samples. For absolute qPCR, we first constructed standard samples for each species of bacteria. The corresponding 16S rRNA V3–V4 region sequences obtained by high-throughput sequencing were synthesized by Tsingke Biology Co., Ltd. (Chengdu, China) and cloned into the pMD^®^ 19-T vector (Takara Biotechnology Co., Ltd., Dalian, China). The vectors were transduced into competent *Escherichia coli* DH-5α cells, aliquots were spread on agar plates, and single colonies were selected. After culturing, plasmids were extracted using the TIANprep Mini Plasmid Kit (Tiangen Biotech Co., Ltd., Beijing, China) following the product manual. The concentration of plasmids was measured, and the copy numbers were calculated according to relative plasmid quality. All plasmids containing the target fragments were diluted by 10-fold gradients (at least five gradients) for qPCR to monitor amplification efficiency and to generate standard curves (Supplementary Table 2).

### Statistical Analysis

For ASV diversity analysis, we used our resampled ASV table at a depth of 12,000 without replacement as a basis. In α-diversity analysis, the richness and evenness of gut microbiota were assessed by calculating the numbers of different species and the Shannon index (Shannon, 1948), respectively. As for β-diversity, both the Bray–Curtis dissimilarity (Beals, 1984) and the unweighted UniFrac distance (Lozupone and Knight, 2005) were used to generate principal component analysis (PCA) plots. Because of the high similarity of the downstream analysis results based on the matrix calculated from the Bray–Curtis dissimilarity and the unweighted UniFrac distance, only the results based on Bray–Curtis dissimilarity were shown in our study (resultant figures based on unweighted UniFrac distance are shown in Supplementary Materials). The statistical analysis of both α-diversity and β-diversity between groups was performed in QIIME 2 with pairwise Kruskal–Wallis and permutational multivariate analysis of variance (PERMANOVA) tests (Zapala and Schork, 2006; Chen et al., 2012), respectively. Hierarchical clustering was performed with the UPGMA algorithm using the *hclust* package in R (v3.5.3) (R Core Team, 2015). The phylogenetic tree was constructed from ASVs using the method described by Callahan et al. (2016). Dendrograms were created using the package, *ape* (Paradis et al., 2004). A random forest classifier (RFC)-supervised learning algorithm was implemented in the *randomForest* package (Breiman, 2001) in R. Models were run using CSS-normalized ASV counts with 1,000 trees, and the OOB estimates of error rates were counted. The linear discriminant analysis (LDA) effect size (LEfSe) (Segata et al., 2011) was performed on the Galaxy/Hutab online platform^4^ based on the ASV table, and the LDA threshold was set at 3.6 between groups. Other statistical analyses were carried out in SPSS 23. The copy numbers of core microbial members were compared using ANOVA (analysis of variance).

## Results

### Breeding Model Queens

We succeeded in producing 15 crossbred queens (AHQs) out of 31 attempts, and 16 ACQs and 16 *A. mellifera* queens (AMQs) were bred in the same place during the same period. Considering the high individual diversity of gut microbiota, all the queen samples were used for high-throughput sequencing except the necessary biological duplicates for qPCR. In summary, 20 midgut and 19 rectal gDNAs (one rectal gDNA sample was removed for failing to meet the quality requirements) were extracted from 20 queen samples (seven of ACQs, six of AHQs, and seven of AMQs) and used for sequencing library preparation, while another 27 midgut and 27 rectal gDNA extracted from 27 queen samples participated in qPCR. To verify the early colonization traits of bacteria in queens’ gut, the queen samples used for high-throughput sequencing were collected after the queens were fed by nurse bees for 5 days. The samples used for qPCR were collected when the queens were 1, 4, and 7 days of age to monitor the abundance variations of core members of queens’ gut microbiota. Notably, in order to get the reciprocal resultant data from the other kind of AHQ (laid in *A. cerana* colony but bred in the AMC), four times efforts have ended and we still failed with 0% acceptance of the reverse crossbreeding experiments.

### Composition and Diversity of Queen’s Gut Microbiota

**FIGURE 2:**
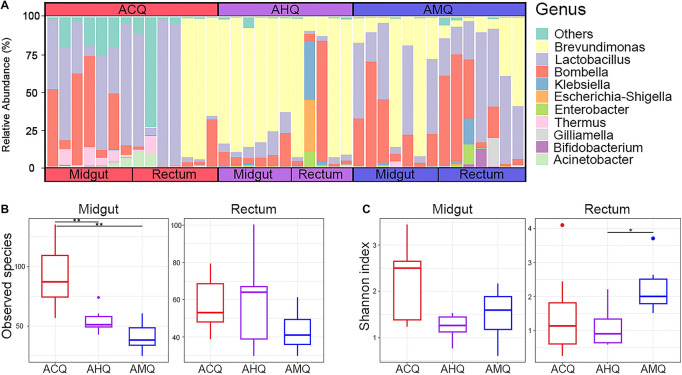
Gut microbiota composition and α-diversity analysis of ACQs, AHQs, and AMQs. **(A)** Bacterial relative abundance of midgut and rectum at the genus level. **(B,C)** The results of observed species and Shannon indexes of α-diversity analysis shown by boxplots colored in red for ACQs, violet for AHQs, and blue for AMQs. *p < 0.05 and **p < 0.01 represent the significance levels between groups calculated from the Kruskal–Wallis *H* test.

Overall, 3,220,523 sequences of the 16S rRNA gene were obtained, forming 1,861,700 ASVs. The number of sequences per sample ranged from 66,247 to 96,700, with an average of 82,578. Our sequencing results showed that the core members of the gut microbiota of queens were different from those of workers. The bacterial community of the three types of queens at the phylum level (Supplementary Figure 1) mainly consisted of Proteobacteria (72.0%) and Firmicutes (26.4%), accounting for over 98% of the total microbial composition. At the genus level (Figure 2A), only five taxa (relative abundance >1.0%) dominated the midgut and rectum bacterial community: *Brevundimonas* (45.0%), *Lactobacillus* (25.4%), *Bombella* (20.6%), *Klebsiella* (2.0%), and *Escherichia–Shigella* (1.2%). Notably, two of seven ACQ rectal samples were completely dominated by a single taxon, like *Lactobacillus*, while other two rectal samples were mainly dominated by *Brevundimonas*. We found that the composition of the rectum microbiota in ACQs showed two patterns, one dominated by *Lactobacillus* and the other by *Brevundimonas*. The observed ASV numbers (Figure 2B) and Shannon index (Figure 2C) were used to assess the richness and evenness, relatively. The number of taxa in the midgut or rectum of ACQs was higher than the other two groups, but the difference was only significant for the midgut (*p* < 0.01). As for the microbial evenness, the gut microbiota of the rectum and midgut in the AHQ group was in the middle level, and only the difference between AHQs with AMQs in the rectum was significant (*p* < 0.05).

### High Similarity of the Midgut Microbiota Between Asexual Hybridization Queens and *Apis mellifera* Queens

**FIGURE 3:**
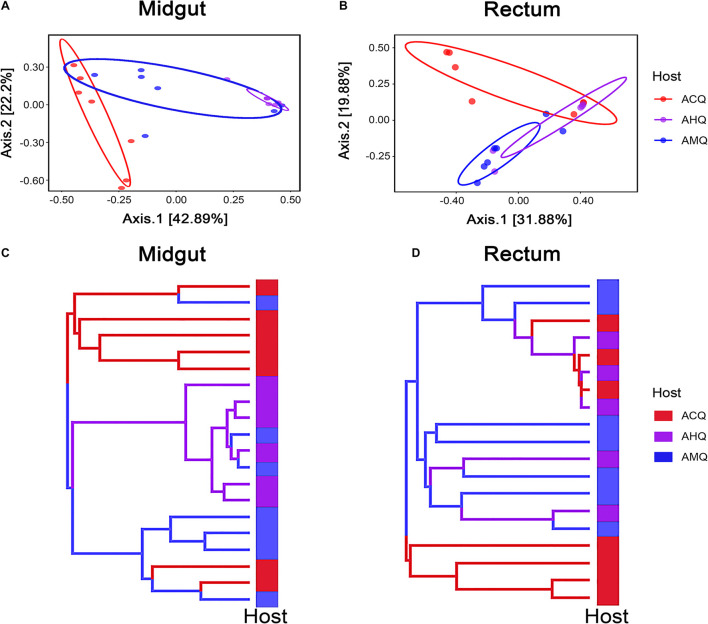
High microbiota similarities between AHQs and AMQs. **(A,B)** β-Diversities of bacterial communities are clustered using principal coordinate analysis (PCoA) based on Bray–Curtis dissimilarity (results of unweighted UniFrac distance are shown in Supplementary Figures 2A,B). Each dot on the plot represents the entire microbiota of a gut tissue sample, and the dots were colored correspondingly. **(C,D)** The dendrograms rebuilt using the UPGMA method based on Bray–Curtis dissimilarity. Each topological branch is also colored the same as before.

**FIGURE 4:**
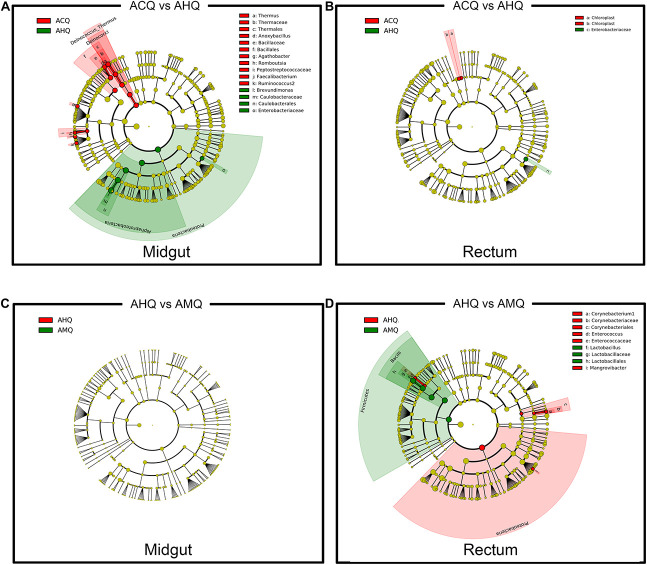
Genetic-specific and environmental-specific taxa filtrating through intergroup comparison. **(A,B)** The differentially abundant taxa of the midgut and rectum comparing ACQs (red) vs. AHQs (green) were identified using LEfSe analysis and displayed in color to detect the genetic-specific taxa. **(C,D)** The other comparison between AHQs (red) and AMQs (green) in the midgut and rectum was also performed to identify the environmental-specific taxa. Each circle diameter is proportional to the taxon abundance.

To explore the deeper connections of the gut microbial community of AHQs and other queens, multiple analyses were performed. After that, we found several evidences that could support the gut microbiota composition of AHQs which was more similar to that of AMQs. Firstly, the principal coordinate analysis (PCoA) plots based on Bray–Curtis dissimilarity showed that the AHQ midgut sample clustering had a stronger correlation with AMQs while the AHQ rectal sample clustering fell between ACQs and AMQs (Figure 3A). Similar patterns were observed in the PCoA plots based on unweighted UniFrac distances (Supplementary Figures 2A,B). Secondly, the topological structure of the UGPMA trees based on Bray–Curtis dissimilarity (Figures 3C,D) and unweighted UniFrac distances (Supplementary Figures 3A,B) showed that both in the midgut and rectum, most AHQs and AMQs clustered into one branch while ACQs appeared in another single branch; the only exception was the rectum topological structure based on unweighted UniFrac distances in which most AHQs were clustered with ACQs (Supplementary Figure 3B). Next, RFC models classified the midgut gut microbial communities from queens’ genetic background (AMQs + AHQs vs. ACQs) with great accuracy (95%) while the classification accuracy according to queens’ environment (AMQs vs. ACQs + AHQs) was poor (70%). Most definitively, LEfSe tests were employed to identify the taxa of significant differences among AHQs and other queens. Considering the relative simplicity of the composition of queens’ gut microbiota and the similarity of core members among different queen types, we initially adjusted the LDA threshold to a relatively high level of 4 to screen out the significant taxa among groups more strictly. As visualized in Figures 4A–D, significant differences in taxa between AHQs and the other two groups in the midgut and rectum were detected and varied greatly in number except the comparison between the AHQs vs. AMQs in the midgut, which showed no significant differences in taxon (Figure 4C). Even when the threshold was lowered to 3.6 (another commonly used threshold), there were still no significant differences in taxon.

Collectively, these studies consistently revealed the high similarity between AHQs and AMQs, indicating that the queens’ genetic background played a more important role than environmental factors in shaping the midgut microbiota. Because AHQs and AMQs were laid by the same queen but bred in different colonies, they had the same genetic background but different nutritional and living environmental conditions. However, attributing all the factors determining the gut microbial composition to genetics would be unrealistic because environmental factors did play a part in the determination. For instance, we could not completely separate the AHQ cluster from the ACQ cluster from the midgut or rectum in the vertical axis of the PCoA plots based on Bray–Curtis dissimilarity (Figures 3A,B). Early feeding or other environmental factors could contribute to the establishment of the microbial community of AHQs.

### Screening for Genetic-Specific Taxa

Based on the results of LEfSe analyses (Figure 4) and RFC models (Supplementary Figure 3), we tried to seek out the specific taxa of strongly genetic preference according to the following two requirements: firstly, either in midgut or rectum, the absolute abundances of these specific taxa must be significantly different between ACQs and AHQs, but not between AHQs and AMQs. This is because ACQs and AHQs only share the same nurturing conditions, but not the same genetic background. Secondly, to make our results more rigorous, these taxa must be identified simultaneously in both LEfSe analyses and RFC models (Supplementary Figure 3). Finally, there were seven taxa were identified from the midgut with high genetic preference. These genera included *Romboutsia*, *Brevundimonas*, *Faecalibacterium*, *Anoxybacillus*, *Thermus*, *Agathobacter*, and *Ruminococcus2*. All of these taxa had a higher abundance in ACQs except *Brevundimonas* in AHQs. As for rectum, only three taxa were figured to have a higher abundance in AHQs, all belonging to Enterobacteriaceae.

Consistent with the results of species composition and α-diversity analysis (Figures 2A–C), these findings all indicated that the development of the gut microbiota of AHQs resulted in lower diversity and dominance of several taxa, especially *Brevundimonas*. In our view, the high abundance of *Brevundimonas* can be explained in two ways. Firstly, *Brevundimonas* could have already colonized the advantageous metabolic niches in the early developmental stage of gut microbiota in AHQs, and its rapid proliferation kept it at high levels throughout the establishment of the gut microbiota community (high absolute and high relative abundance). The other explanation could be that it was not the rapid proliferation of *Brevundimonas*, but a slower growth rate of other bacteria, making the dominance of *Brevundimonas*.

### Early Developmental Patterns of the Core Members

**FIGURE 5:**
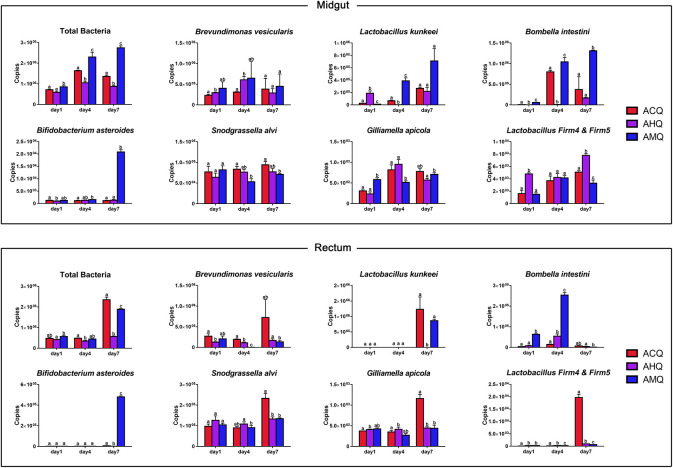
Changes of core members’ absolute abundance in queens’ early developmental stage (1, 4, and 7 days) measured as copies of the 16S rRNA gene. The columns of host groups were differently colored in red for ACQs, violet for AHQs, and blue for AMQs. Letters above confidence intervals (1 standard deviation) represent significance levels (Tukey’s HSD).

To explore the developmental patterns of the core members of queens’ gut microbiota in host early development, we used qPCR to measure the absolute abundance of total bacteria and of the seven main taxa (including three dominant taxa of queens and four core members of workers) in three groups of 1-, 4-, and 7-day-old queens (parameters of the resultant standard curve are listed in Supplementary Table 2). In general, specific bacteria exhibited different proliferation patterns among the different queens (Figure 5). The time at which the absolute abundance of specific bacteria reached the highest varied among different queens and in different gut sections. The total bacteria in the midgut of AMQs remained the highest throughout the host’s early development stage, followed by ACQs, and the total number of bacteria was the lowest in AHQs (*p* < 0.05). The total bacteria in the other two groups showed a decreased rate of proliferation, while bacteria in AMQs maintained a high growth rate. The situation in the rectum was reversed in 7-day-old queens. The bacteria in ACQs proliferated explosively from day 4 to day 7, when it reached the highest, surpassing the numbers in the AMQs (*p* < 0.05). Inexplicably, the number of total bacteria from AHQs remained at a low level during the early developmental stage and showed no growth trend. Changes in absolute abundance of *Brevundimonas* (genetic-specific taxon) in AHQs showed the same pattern of development as in AMQs: high abundance but slow growth rate in the midgut and consistently low abundance in the rectum. However, the other two dominant taxa (*Lactobacillus kunkeei* and *Bombella*) showed unique growth patterns, different from both AMQs and ACQs, and maintained low abundance levels throughout, which were consistent with the results of high-throughput sequencing. Lastly, relatively stable development patterns of the core members of workers were observed in the guts of queens while only a few species of bacteria had extremely high colonization in specific gut sections, like *Bifidobacterium asteroides* and *Lactobacillus Firm4* and *Firm5* in the rectum, indicating differences between core members in the gut microbiota of queens and workers.

## Discussion

Overall, our high-throughput sequencing and absolute qPCR results suggest that the rate of core bacterial proliferation of the three groups of queens varied greatly at each time point. The core genera were *Brevundimonas*, *Lactobacillus*, and *Bombella*, and the major phyla were Proteobacteria and Firmicutes. These results differed from the results with adult *A. mellifera* queens in which the core members included *L. Firm5* (51.3%), *Parasaccharibacter apium* (*Bombella*) (27.1%), and *L. kunkeei* (7.6%) (Anderson et al., 2018; Powell et al., 2018), especially for *Brevundimonas*, which were hardly ever reported. The reason for the difference may be attributed to the age of the queen at sampling as the gut changes with age. The gut microbiota of queens was dynamically changing, and that is why we focused on comparing the early establishment of core members between different queen types. It was worth noting that verifying the gut microbiota of adult and mated queens is equally valuable yet unsuitable for exploring the determinant of the gut microbiota of queens in their early development stage. Therefore, this study was only limited to the perspective of the study on the valuation of the gut microbial community in queens’ early stage.

*Apis mellifera* and *A. cerana* belong to the same genus, but they are quite different in morphology and anatomy (Li et al., 2012) and also in their gut microbiota. In our results, the composition of the gut microbiota of AHQs and AMQs was relatively stable and predictable while the relative abundance of certain taxa varied in each ACQ sample. In most AHQ and AMQ samples, the main microbial members accounted for more than 95% of the total, while the ACQ samples were variable. Interestingly, *Brevundimonas*, a non-core member of the gut microbiota of workers, was almost dominant in the gut microbiota of AHQ and AMQ samples and could also be detected in ACQ rectum samples. This suggests that the initial rectal microbial community of ACQ may have two types, one dominated by *Lactobacillus* and the other by *Brevundimonas*. In terms of microbial composition, the AMQ’s gut microbes were more likely to be the further developmental model of AHQ. In this model, the relative abundance of *Brevundimonas* decreased gradually with increasing age of the host while the proportion of other core members increased gradually. The composition of the gut microbiota changed from a single taxon predominating to one in which there were multiple core members dominating the totals. As a result, we speculated, the functional roles of the bacterial community gradually improved and interaction with the host increased. Based on the data available, we suggested that the composition of the gut microbial community was basically determined by the queens’ genetic background, while environmental factors influenced the rate of improvement of the gut microbial community. In our study, all non-genetic factors were generally combined as environmental factors. However, the environmental factors were actually composed of a variety of different elements. The weight of each factor should be taken into account when further studying the influence of the environment on the microbiota.

Unfortunately, our small sample size may be the major limitation to reach conclusions with high confidence. Although our sample number reached the bottom line of statistical significance, the differences of gut microbiota among organisms were prone to be amplified by a variety of uncertain factors, which further influenced our observations. Moreover, we also tried to transfer the *A. cerana* queen pupae to young AMC to breed the other kind of crossbred queen, which could give the other important reciprocal evidence to support our main conclusion robustly. Unfortunately, four times efforts have ended with 100% failure acceptance (all *A. cerana* fertilized eggs were cleaned out from *A. mellifera* hives), which may be because *A. mellifera* workers had a higher discriminability for other honeybee species than *A. cerana* workers, and these alien individuals were excluded completely as previous studies reported (Rinderer et al., 1985; Robinson, 1987; Ruttner, 1988). The strong rejection of *A. mellifera* to other bee species hinders the applications of crossbreeding, hoping that this hindrance can be removed in the future.

The gut of the honeybee consists of the midgut, ileum, and rectum. The midgut was the main organ for absorbing water and digesting sugars and other nutrients. There were very few bacteria in the midgut of honeybees, which may be because the midgut is unable to provide a stable venue for the establishment and proliferation of bacteria (Martinson et al., 2012; Corby-Harris et al., 2014a). In contrast, the highly anaerobic environment and the stable substrate supply gave the rectum region the greatest concentration of bacteria (Kapheim et al., 2015; Ludvigsen et al., 2015; Jones et al., 2017; Yun et al., 2018). However, our qPCR results showed that the absolute abundance of total bacteria in the midgut was higher overall than that in the rectum, which was contrary to former studies, yet the rate of growth of total rectal bacteria increased sharply between the fourth and seventh days. Together, these results implied that the rectal microbial community was in a rapid development phase and its abundance would soon exceed the microbiota in the midgut at one time point in the future. We found that detecting the significant taxa in the midgut was easier than in the rectum when we tried to screen out genetic-specific taxa, which was also supported by β-diversity analysis results. By comparing α-diversity, we found that both the microbial richness and the variation in evenness of the midgut were greater than in the rectum. In general, these results suggested that the developmental direction of gut microbiota in the midgut and rectum appeared different. The midgut was more likely to show the influence of genetic background during the early life stage of the queens.

In summary, our results from multiple analyses indicated that the genetic background played a more dominant role than environmental factors in shaping the microbiota of the midgut. Compared to the microbial community of the midgut, the rectal microbiota was more insensitive to the impact of genetic background during the early developmental stage of honeybee queens.

## Data Availability Statement

The datasets presented in this study can be found in online repositories. The names of the repository/repositories and accession number(s) can be found below: https://www.ncbi.nlm.nih.gov/ PRJNA751765.

## Author Contributions

JY and MY designed the study. YuZ and LX collected the samples and data. KL and JY was the project administration. YeZ performed the laboratory analyses while LX and BZ performed the bioinformatics analyses on software. MZ and DiL funded this study. YuZ, MY, and DeL wrote the original draft of manuscript together. All authors contributed to the article and approved the submitted version.

## Conflict of Interest

The authors declare that the research was conducted in the absence of any commercial or financial relationships that could be construed as a potential conflict of interest.

## Publisher’s Note

All claims expressed in this article are solely those of the authors and do not necessarily represent those of their affiliated organizations, or those of the publisher, the editors and the reviewers. Any product that may be evaluated in this article, or claim that may be made by its manufacturer, is not guaranteed or endorsed by the publisher.

## Footnotes

1
http://ccb.jhu.edu/software/FLASH/

2
https://qiime2.org/

3
https://www.arb-silva.de/

4
http://huttenhower.sph.harvard.edu/galaxy/

## Supplementary Material

Supplementary Figure 1 The barplot of the composition of queens’ gut microbial community in phylum level.

Supplementary Figure 2 **(A)** The principal co-ordinates analysis (PCoA) plot based on the unweighted UniFrac matrix of queens’ midgut microbiota. **(B)** The principal co-ordinates analysis (PCoA) plot based on the unweighted UniFrac matrix of queens’ rectum microbiota.

Supplementary Figure 3 The dendrograms rebuilt using UPGMA method based on unweighted UniFrac distance.

Supplementary Figure 4 The top 45 diverse taxa detected by RFC models.
