# Supplementary figures and images for: The Dominating Role of Genetic Background in Shaping Gut Microbiota of Honeybee Queen Over Environmental Factors
Source: Front Microbiol. 2021 Nov 5;12:722901. doi: 10.3389/fmicb.2021.722901 (PMC8603915; doi:10.3389/fmicb.2021.722901)

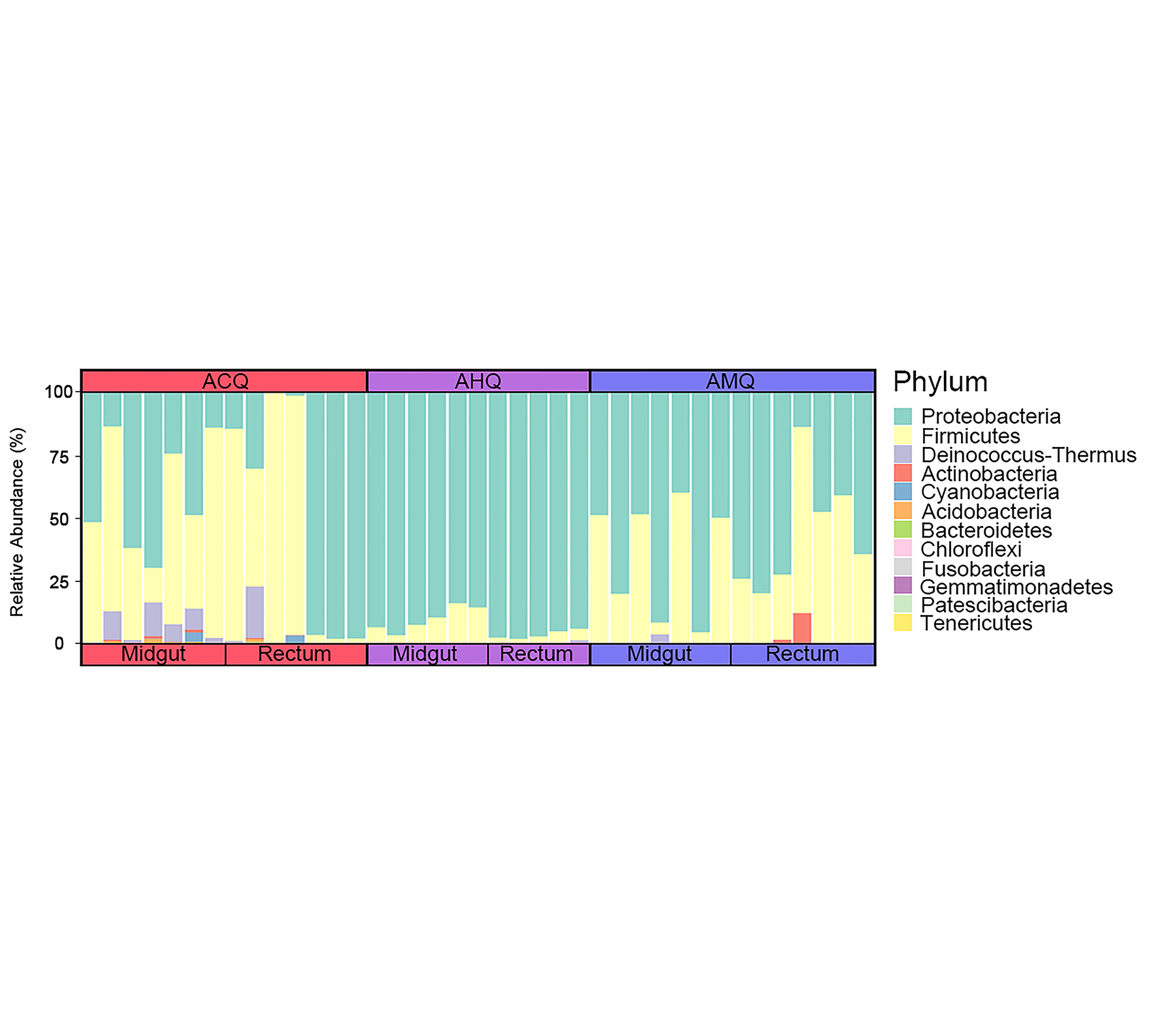

Supplement: Supplementary Figure 1 — The barplot of the composition of queens’ gut microbial community in phylum level. [file Image_1.TIF]

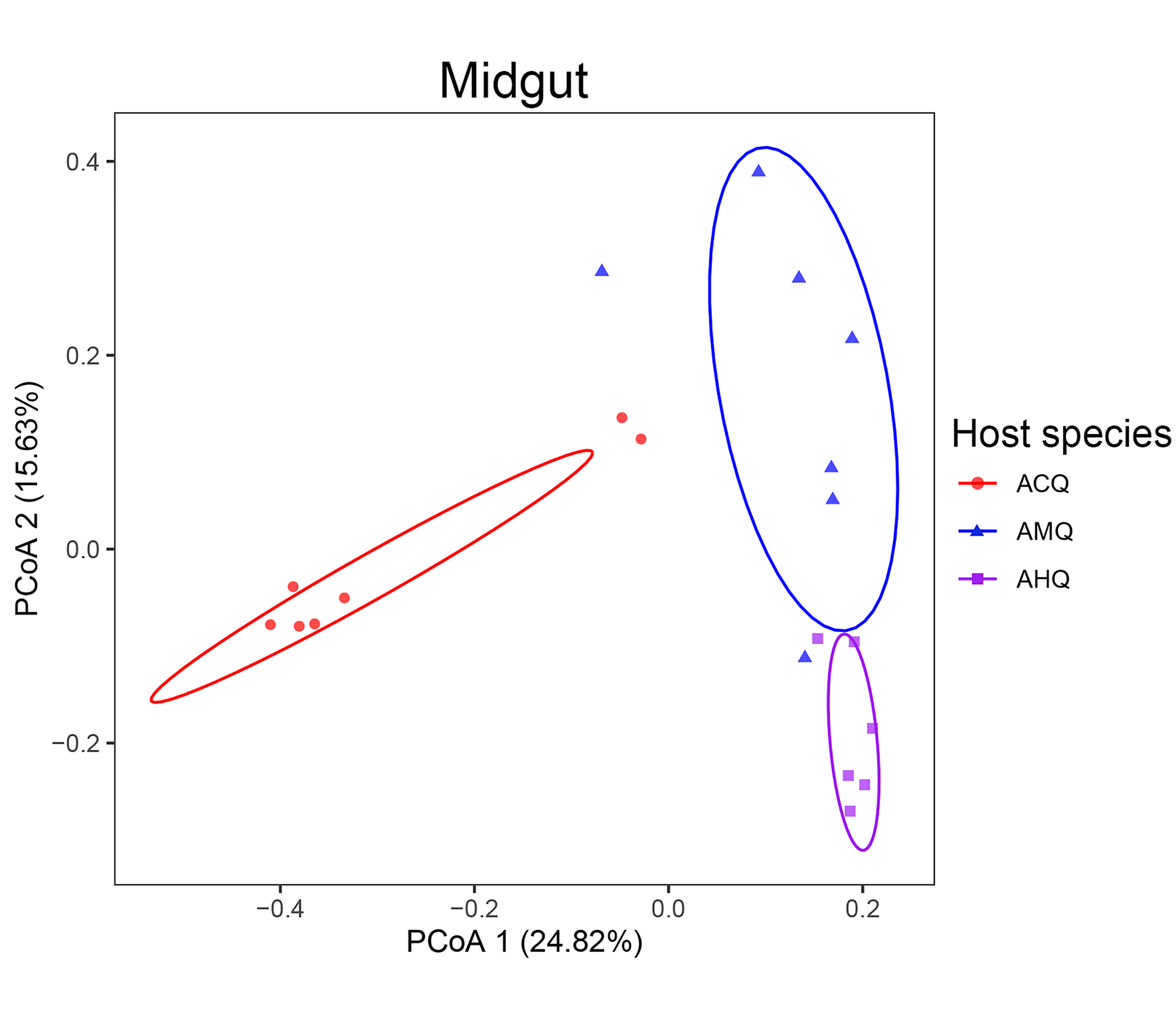

Supplement: Supplementary Figure 2 — (A) The principal co-ordinates analysis (PCoA) plot based on the unweighted UniFrac matrix of queens’ midgut microbiota. (B) The principal co-ordinates analysis (PCoA) plot based on the unweighted UniFrac matrix of queens’ rectum microbiota. [file Image_2.TIF]

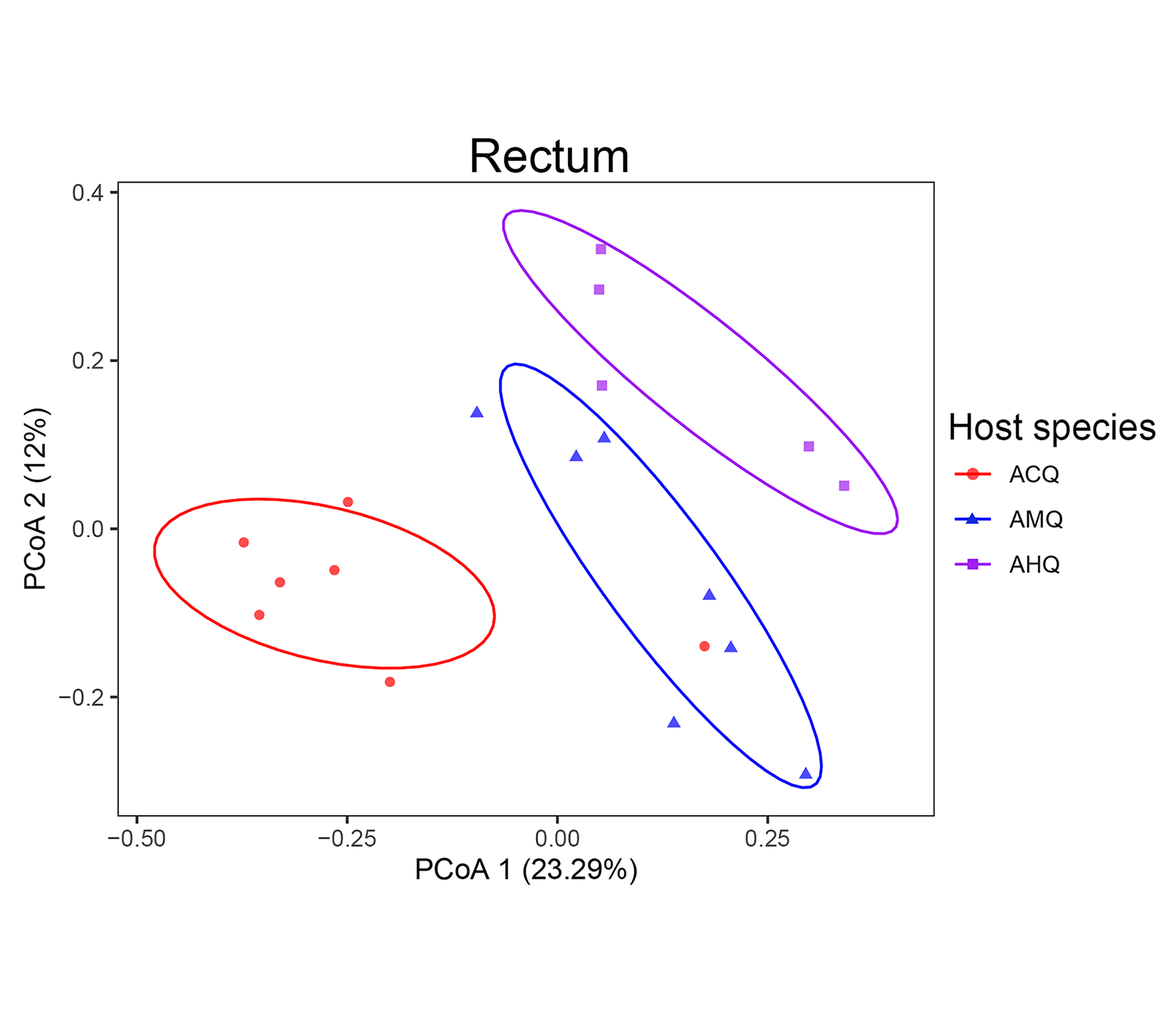

Supplement: Supplementary Figure 3 — The dendrograms rebuilt using UPGMA method based on unweighted UniFrac distance. [file Image_3.TIF]

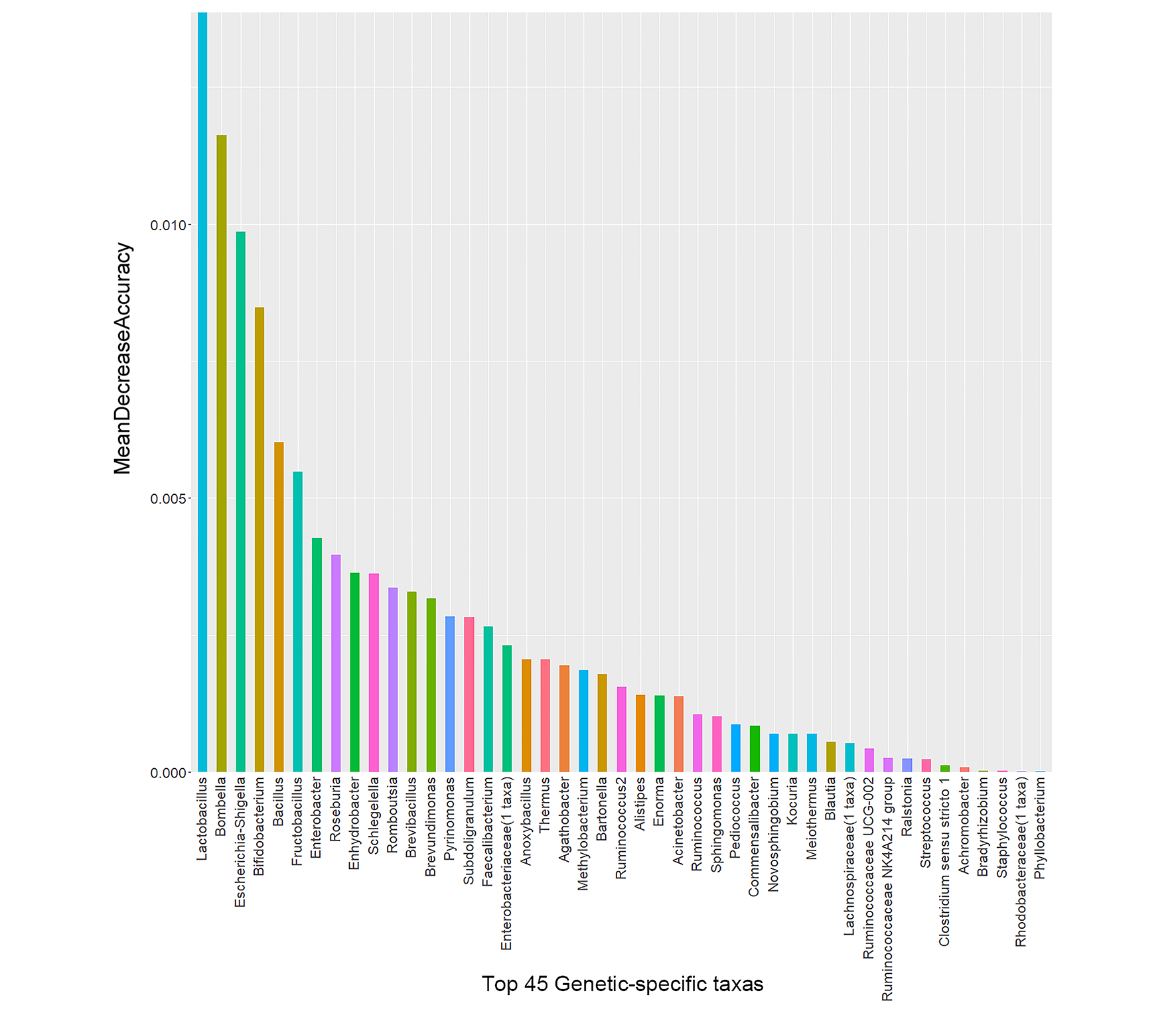

Supplement: Supplementary Figure 4 — The top 45 diverse taxa detected by RFC models. [file Image_4.TIF]

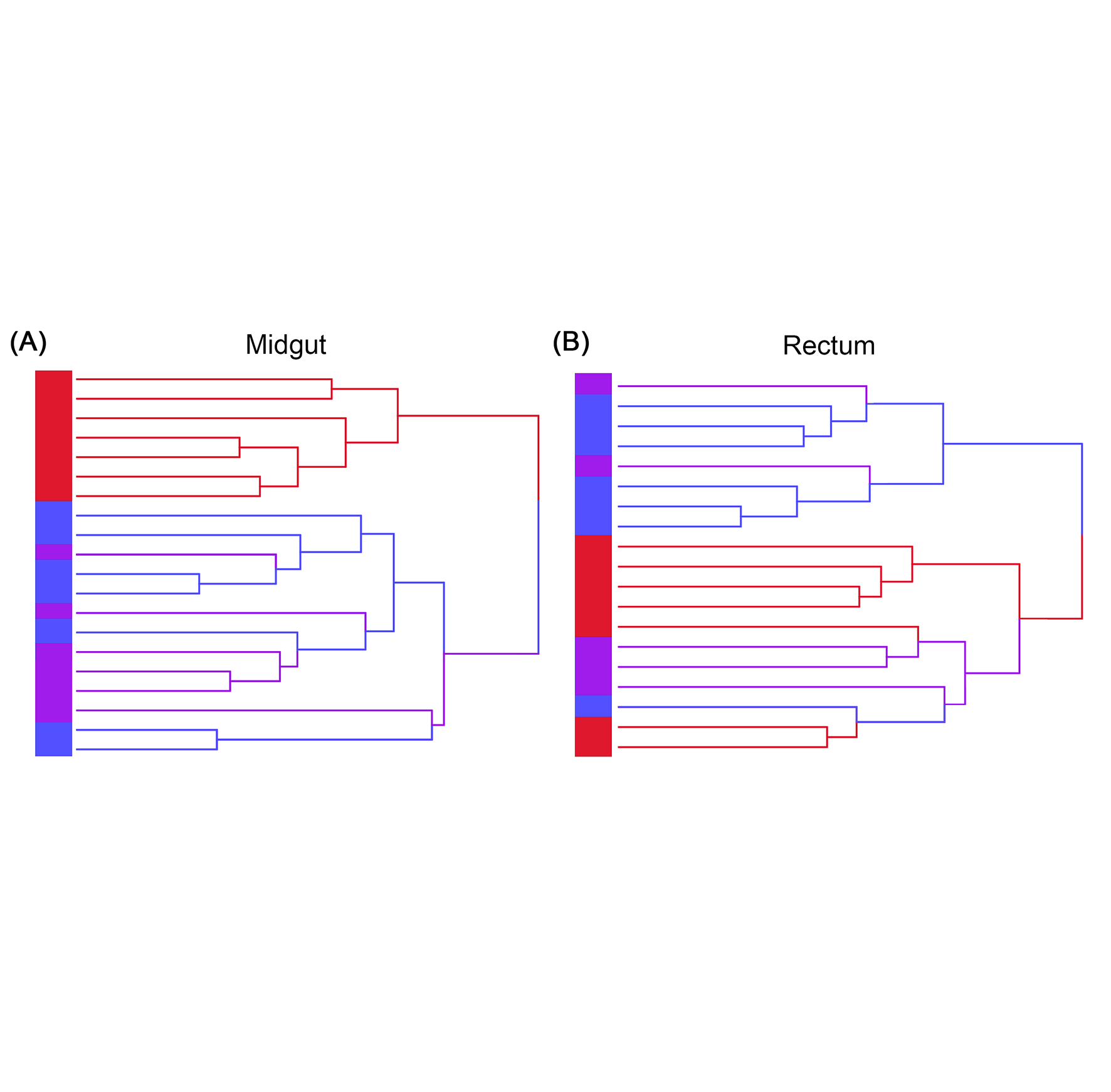

Supplement: Supplementary file 5 [file Image_5.TIF]
